# Manipulating interstitial carbon atoms in the nickel octahedral site for highly efficient hydrogenation of alkyne

**DOI:** 10.1038/s41467-020-17188-3

**Published:** 2020-07-03

**Authors:** Yiming Niu, Xing Huang, Yongzhao Wang, Ming Xu, Junnan Chen, Shuliang Xu, Marc-Georg Willinger, Wei Zhang, Min Wei, Bingsen Zhang

**Affiliations:** 1 Shenyang National Laboratory for Materials Science, Institute of Metal Research, Chinese Academy of Sciences, 110016 Shenyang, China; 2 Department of Materials Science and Engineering, University of Science and Technology of China, 230026 Hefei, China; 3 Department of Inorganic Chemistry, Fritz Haber Institute of the Max Planck Society, 14195 Berlin, Germany; 4 Scientific Center for Optical and Electron Microscopy, Otto-Stern-Weg 3, ETH Zurich, 8093 Zurich, Switzerland; 5 State Key Laboratory of Chemical Resource Engineering, Beijing Advanced Innovation Center for Soft Matter Science and Engineering, Beijing University of Chemical Technology, 100029 Beijing, China; 6 Dalian National Laboratory for Clean Energy, Dalian Institute of Chemical Physics, Chinese Academy of Sciences, 116023 Dalian, China; 7 Electron Microscopy Center, Key Laboratory of Automobile Materials MOE, and School of Materials Science & Engineering, Jilin University, 130012 Changchun, China

**Keywords:** Catalyst synthesis, Heterogeneous catalysis, Nanoparticles, Transmission electron microscopy

## Abstract

Light elements in the interstitial site of transition metals have strong influence on heterogeneous catalysis via either expression of surface structures or even direct participation into reaction. Interstitial atoms are generally metastable with a strong environmental dependence, setting up giant challenges in controlling of heterogeneous catalysis. Herein, we show that the desired carbon atoms can be manipulated within nickel (Ni) lattice for improving the selectivity in acetylene hydrogenation reaction. The radius of octahedral space of Ni is expanded from 0.517 to 0.524 Å via formation of Ni_3_Zn, affording the dissociated carbon atoms to readily dissolve and diffuse at mild temperatures. Such incorporated carbon atoms coordinate with the surrounding Ni atoms for generation of Ni_3_ZnC_0.7_ and thereof inhibit the formation of subsurface hydrogen structures. Thus, the selectivity and stability are dramatically improved, as it enables suppressing the pathway of ethylene hydrogenation and restraining the accumulation of carbonaceous species on surface.

## Abstract (web summary)

Design and synthesis of non-noble metal catalysts is crucial for highly-efficient hydrogenation. Here, the interstitial sites in nickel are manipulated with zinc and filled by dissociated carbon atoms, which drastically improve the selectivity/stability in acetylene hydrogenation reaction.

## Introduction

Interstitial sites of transition metal catalyst are found to be occupied by dissociated atoms under a high chemical potential of reactant molecules^1–4^. Definitely, the interstitial atoms enable modifying electronic/geometric properties of surface atoms, which directly affects its Fermi level and density of states. Thus, the catalytic behaviors were tuned. For instance, the subsurface O in Cu nanoparticles (NPs) (~10 nm) impairs a positive charge to the Cu surface and thereof improves the catalytic performance in methanol steam reforming reaction^5^. More interestingly, the interstitial atoms are clarified to directly participate in reactions and afford the distinct reaction pathways from the surface-adsorbed atoms. So it leads to diverse products, reaction kinetics, and mechanisms. As evidenced by Ceyer et al.^2^, the interstitial H in Ni subsurface is found to be active for hydrogenation adsorbed CH_3_ to CH_4_ whereas the surface-adsorbed H remains unreactive with CH_3_ species. Initially, the interstitial site manipulated catalysis is considered to affect a few metal catalysts and associated reactions. More transition metal-catalyzed reactions, however, are controlled by the generation of metastable interstitial atoms^6–9^. Thus, it is of great interests to explore the control of interstitial site atoms in transition metal catalysts for tuning their catalytic performances^10–12^.

Acetylene selective hydrogenation, a well-known industrial reaction, with which a switchable selectivity towards distinct products is determined by the alternation of interstitial atoms in Pd catalyst^1,13–15^. Experimental studies demonstrated that carbon or hydrogen atoms in the interstitial sites of Pd subsurface govern the selectivity to alkene or alkane^1,16^. Similar results were also observed in Ni, which is widely adopted as industrial hydrogenation catalyst (e.g., Raney Ni) for high activity and low cost in other heterogeneous reactions. However, it suffers from the drawbacks of low selectivity and stability in alkyne selective hydrogenation reaction^17,18^. Some investigations demonstrate that interstitial H atoms can readily hydrogenate adsorbed C_2_H_4_ to C_2_H_6_. It drives C_2_H_4_ from underneath with an orientation to the rehybridized π orbitals, although the surface H atoms remain unreactive^19^. These results reveal that it is the occupation of interstitial sites with carbon atom that is responsible, instead of hydrogen atoms, in Ni group catalysts. Following this behavior, the selectivity would be improved in hydrogenation. Thus, the formation of subsurface interstitial atoms was thereof focused on. Unfortunately, it is highly kinetically dependent and suffers from an input of external energies to stabilize; it remains challenging for the atomic-level control of desired atoms^14,20^. The common strategy to stabilize the metastable interstitial structure lies at construction of an encapsulated shell. Following this approach, PdC_x_@C structure was successfully prepared with the removal of the graphite layers after external procedures^21^. However, it is even difficult for the formation of Ni-based interstitial carbon structure because of the usually required high temperatures. The dissolved carbon atoms within Ni lattices are readily segregated on the surface. As a result, it offers the formation of graphite layers or even carbon nanotubes/nanofibers (CNTs/CNFs) under reaction conditions. Following the removal of carbon shells is even more challenging, during which Ni is readily oxidized and the interstitial carbon structure is damaged due to the oxyphilic nature of Ni. That is to say, it is crucial to modulate Ni environments in order to accommodate carbon atoms with directly exposed active surfaces instead of applying external process.

In general, the interstitial atoms prefer to locate in octahedral hole than at tetrahedral sites in view of the larger space with more available coordination atoms^22^. The radius of octahedral (*R*_oct_) site in face-centered cubic (FCC) metals can be calculated by following the equation *R*_oct_ = 0.414*R*_M_ (*R*_M_ represents the metallic radius of metal atom)^23^. Compared with Pd (0.569 Å), the smaller *R*_oct_ in Ni (0.517 Å) leads partially to both a higher carbon dissolving temperature and an easier segregated carbon atom on surface. Therefore, a structural transformation from Ni (FCC) to Ni_3_C (hexagonal close-packed structure) occurs to accommodate a certain amount of carbon atoms, together with uncontrollable formation of graphitic layers^24,25^. Theoretical studies have evidenced that the expansion of metal lattice parameters leads readily to inclusion and diffusion of atoms at interstitial sites^26–28^. Second metal introduction can effectively tune the lattice parameter of Ni^29,30^. In addition, the electronic structure should not be changed in order to maintain the capability of Ni to dissociate adsorbed molecules. In Ni–Zn systems a Zn addition may expand the lattice parameters of Ni and generally leave a slightly modified electronic structure^30,31^.

Herein, *R*_oct_ increases from 0.517 to 0.524 Å, provided if 25 at% Zn is introduced to Ni to form Ni_3_Zn, with a marginal change of electronic structure. Simple impregnation method and hydrogen/acetylene treatment (the procedures are illustrated in Supplementary Fig. 1) readily generate the interstitial carbon structure Ni_3_ZnC_0.7_ at low temperature, characterized by integrating in situ and ex situ techniques. It demonstrates that the supported Ni_3_ZnC_0.7_ catalyst exhibits high selectivity at different ratios of H_2_/alkyne and an excellent stability in acetylene hydrogenation reaction. The dramatic improvement is ascribed to two issues: the interstitial carbon modulation of Ni surface/subsurface structures and the suppressed accumulation of carbonaceous fragments on the surface.

## Results

### Incorporation of carbon atom into Ni

**Fig. 1:**
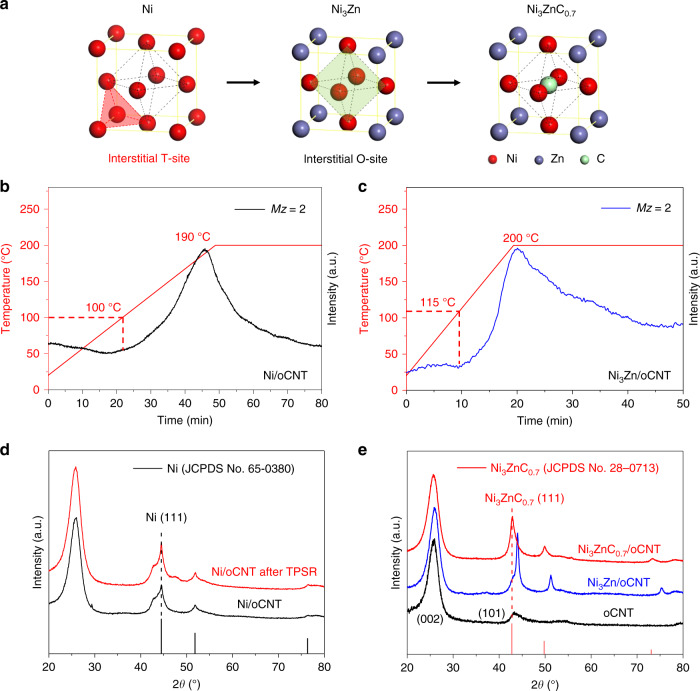
Incorporation of carbon atom in Ni_3_Zn. **a** Schematic of expanded interstitial sites in the unit cells of Ni, Ni_3_Zn, and Ni_3_ZnC_0.7_. The red and black dash lines indicate the tetragonal and octahedral interstitial sites. TPSR-MS results of Ni/oCNT (**b**) and Ni_3_Zn/oCNT (**c**) at elevated temperatures under 1.0 vol% C_2_H_2_/He atmosphere. XRD patterns of Ni/oCNT (**d**) and Ni_3_Zn/oCNT (**e**) after reduction and acetylene treatment.

Carbon materials with abundant defects, since providing additional chemical potential of carbon, are selected to probably diffuse and stabilize carbon in the interstitial sites of supported Ni-based structure^32^. In order to well disperse Ni and Zn ions, oxidized carbon nanotube (oCNT) is optimized for its high surface area and adjustable homogeneous functional sites^33^. Other generally adopted supports such as high-surface-area Al_2_O_3_ is also an ideal candidate, but the desired structure has been not obtained (Supplementary Fig. 2). For the preparation process, Ni or stoichiometric Ni/Zn salts were uniformly impregnated on oCNT (Supplementary Fig. 3) and subjected to a temperature ramp under hydrogen atmosphere. During the reduction process, Ni promoted the reduction of Zn ions and contributed to the formation of Ni_3_Zn structure at 500 °C under hydrogen atmosphere, as evidenced by in situ X-ray diffraction (XRD) experiments in Supplementary Fig. 4. A supported Ni catalyst was also reduced at 500 °C for reference. The lattice parameter of Ni increased from 3.562 to 3.582 Å via the formation of Ni_3_Zn structure, as shown by the diffraction peak shifting from 44.5° to 43.7° of (111) plane in XRD patterns (Supplementary Fig. 5). The radius of octahedral space is expanded from 0.517 to 0.524 Å (Fig. 1a) calculated by using the aforementioned equation.

Previous studies based on Ni–ZnO system have revealed that the formation of Ni–Zn alloy does not change the electronic structure of Ni much^31^. In addition, theoretical calculation exhibits that there is little hybridization between Ni 3*d* and Zn 3*d* states in Ni_3_Zn^30^. Thus, it seems that the electronic structure and the capability to dissociate hydrocarbon molecules of Ni have merely altered in Ni_3_Zn structure, as it is the initial step to form the interstitial carbon structure. Therefore, temperature-programmed surface reaction (TPSR) experiments were conducted to determine the dissociation temperature of acetylene on the surface of Ni and Ni_3_Zn. Acetylene was adopted in that it is the most reactive carbon source due to its highest change of Gibbs free energy in forming metal carbide structure^34^. The results in Fig. 1b, c exhibit that Ni_3_Zn structure maintain the strong capability to dissociate acetylene with an only 15 °C delay compared to Ni. Although Ni exhibits the strong capability to dissociate acetylene, the XRD results (Fig. 1d) revealed that the Ni NPs maintained in the FCC structure after 1.0 vol% C_2_H_2_/He treatment until 500 °C. The dissociated carbon atoms tend to segregate from the Ni NPs and form graphitic layers or even CNTs/CNFs instead of the stable carbides. It has been observed that instead of Ni carbide formation, it is carbon encapsulation that prefers to occur to Ni carbide formation in Ni NPs supported on oxidized CNTs (Ni/oCNTs). TEM investigations confirm the encapsulation of graphitic layers on the FCC Ni NPs (Supplementary Fig. 6). The morphology of Ni NPs is greatly changed after the acetylene treatment, which is well consistent with the reported dynamic behavior and elongation of Ni NPs during CNT growth^35^. Ni_3_Zn exhibits a slightly reduced capability to dissociate acetylene in comparison with Ni. Interestingly, the carbon atom could readily dissolve and diffuse in Ni_3_Zn and form the Ni_3_ZnC_0.7_ structure at low temperature under 1.0 vol% C_2_H_2_/He atmosphere. The lattice parameter of Ni_3_Zn is further enlarged with the carbon inclusion. As indicated by the diffraction peaks in XRD patterns (Fig. 1e), they shift left and also consistent well with Ni_3_ZnC_0.7_ structure (*a* = *b* = *c* = 3.66 Å, JCPDS No. 28-0713).

### Microstructural clarification of Ni_3_Zn and Ni_3_ZnC_0.7_

**Fig. 2:**
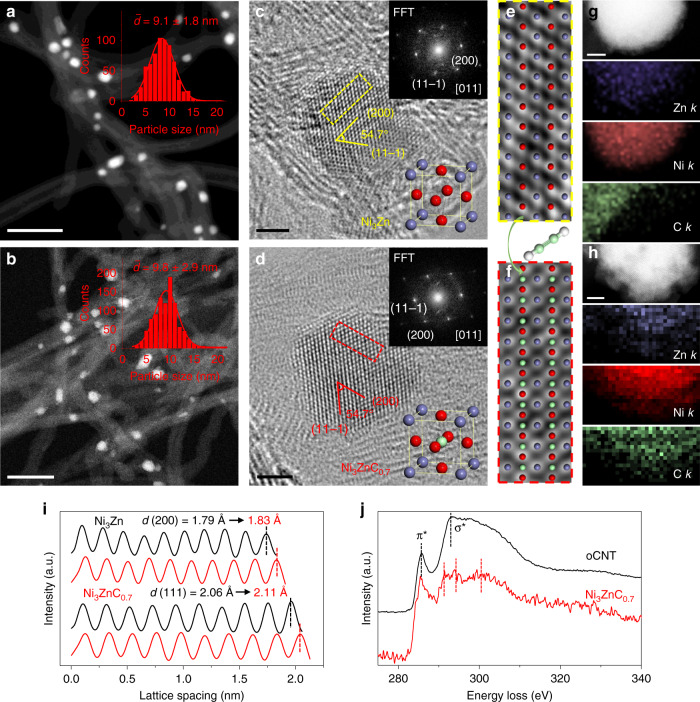
Microstructural characterization of Ni_3_Zn and Ni_3_ZnC_0.7_. STEM images of Ni_3_Zn/oCNT (**a**) and Ni_3_ZnC_0.7_/oCNT (**b**), the right-top insets are the corresponding particle size distribution diagrams. HRTEM images of Ni_3_Zn (**c**) and Ni_3_ZnC_0.7_ (**d**). The top-right and bottom-right insets correspond to the FFTs and unit cells. **e**–**f** The atomic model overlapped with the HRTEM images acquired from the dashed box in **c**, **d**. High-resolution STEM images of Ni_3_Zn (**g**) and Ni_3_ZnC_0.7_ (**h**) with the corresponding EDX elemental mapping of Ni (red), Zn (purple), and C (green). **i** The integrated intensities of Ni_3_Zn and Ni_3_ZnC_0.7_ perpendicular to the (11−1) and (200) planes. **j** C K-edge EEL spectra of oCNT and Ni_3_ZnC_0.7_. Scale bars: 50 nm in **a**, **b** and 2 nm in **c**, **d**, **g**, **h**.

The crystal structures of Ni_3_Zn alloy were identified through XRD measurements: it has the expanded interstitial sites and the generated Ni_3_ZnC_0.7_ structure with carbon incorporation. Following the microstructural features before and after carbon atom inclusion, an aberration-corrected TEM equipped with energy-dispersive X-ray spectroscopy (EDX) elemental analysis and electron energy loss spectroscopy (EELS) is applied. Firstly, high-angle annular dark-field scanning transmission electron microscopy (HAADF-STEM) images demonstrate the morphology of the catalysts. As shown in Fig. 2a, the formed Ni_3_Zn NPs are uniformly distributed on oCNT and exhibit an average particle size of 9.1 ± 1.8 nm. Through the dissociation of adsorbed acetylene and the dissolution of carbon atoms in the Ni_3_Zn structure, the particle size of Ni_3_ZnC_0.7_ NPs increased slightly (9.8 ± 2.9 nm) (Fig. 2b). High-resolution TEM images and corresponding fast Fourier transform (FFT) (Fig. 2c, d) demonstrate the single crystalline nature of NPs in both pristine Ni_3_Zn and Ni_3_ZnC_0.7_. For the characteristic angles of FCC metals, both of which have an acute angle of 54.7° between (200) and (11−1) lattice planes (also illustrated by Fig. 2c, d). An obvious difference in terms of crystal structure lies at the expanded lattice parameters of Ni_3_ZnC_0.7_ after the incorporation of carbon atoms into Ni_3_Zn as evidenced by the aforementioned XRD results. Thus, the spacings of the lattice fringes are compared for (111) and (200) planes of both Ni_3_Zn and Ni_3_ZnC_0.7_ (Fig. 2e, f) collected from either [011] (Fig. 2c, d) or [001] (Supplementary Fig. 7) directions, respectively. The integrated pixel intensities are measured on the basis of the average lattice spacing over ten atomic layers (Fig. 2i). As a result, the average pristine Ni_3_Zn (111) spacing is measured as 2.06 Å, which is consistent with previously reported calculations for Ni_3_Zn^29,30^. The intensity profile of the Ni_3_ZnC_0.7_ atomic layers indicates a larger spacing (2.11 Å) compared with the pristine Ni_3_Zn. Similar integrated pixel intensities are determined for the average lattice spacing of (200) from the [001] direction. To further prove the successful incorporation of the interstitial carbon atoms into Ni_3_Zn, the EDX elemental maps are conducted to provide composition and elemental distribution information. Both Ni (K-edge) and Zn (K-edge) elemental maps of Ni_3_Zn and Ni_3_ZnC_0.7_ NPs are well consistent with the outlines of STEM images (Fig. 2g, h). That is to say, a homogeneous distribution of Ni and Zn existed. Carbon (K-edge) EDX maps in Fig. 2h reveal the existence and uniform distribution of C in Ni_3_ZnC_0.7_ NPs. It is different from Ni_3_Zn structure (Fig. 2g), and explicitly evidence the incorporation of carbon atom after acetylene treatment. The proportions of the atomic ratio in the Ni_3_ZnC_0.7_ NPs are also confirmed via EDX line scan analysis (Supplementary Fig. 8). Subsequently, the body-centered carbon atom is identified by using EELS. The carbon K-edge EEL spectrum of Ni_3_ZnC_0.7_ (Fig. 2j) exhibits three peaks in a range from 290 to 300 eV (291, 294, and 300 eV). Thus, it reveals a unique coordination structure of carbon atom and distinct from the *sp*^2^ structure of carbon in the oCNTs. EELS signal of carbon is not found for Ni_3_Zn NP (Supplementary Fig. 9), which is consistent with the EDX results.

### Catalytic performance in acetylene hydrogenation

**Fig. 3:**
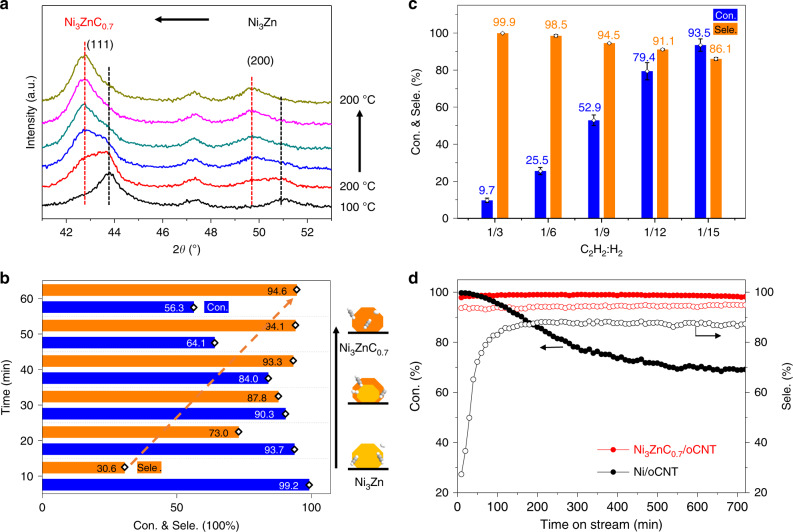
Catalytic performance in acetylene selective hydrogenation reaction. **a** In situ XRD patterns of the structural evolution from Ni_3_Zn to Ni_3_ZnC_0.7_ (black curve: 100 °C, other curves: 200 °C with prolonged times) under a 1.0 vol% C_2_H_2_/He atmosphere. **b** The reactivity corresponds to the structural evolution of Ni_3_Zn/oCNT catalyst under reaction conditions. **c** Conversion and selectivity of Ni_3_ZnC_0.7_/oCNT catalyst (5.0 mg) under various acetylene to hydrogen ratios. Error bars represent standard deviation of experimental data acquired from three times. **d** Stability test (4.5 vol% H_2_, 20.0 vol% C_2_H_4_, 0.5 vol% C_2_H_2_, helium as balance) of Ni/oCNT (15.0 mg) and Ni_3_ZnC_0.7_/oCNT (10.0 mg) catalysts. The solid and hollow symbols represent the acetylene conversion and the selectivity to ethylene.

Microscopic and spectroscopic results explicitly demonstrate that carbon atoms penetrate and occupy the interstitial site of Ni_3_Zn. It is supposed to inhibit the hydrogenation pathway of interstitial hydrogen and increase the selectivity towards ethylene. Thus, the selectivity is detected toward ethylene in acetylene hydrogenation pathway. Meanwhile, the phase transition process from Ni_3_Zn to Ni_3_ZnC_0.7_ is monitored via an in situ XRD experiment (Fig. 3a) under a 1.0 vol% C_2_H_2_/He atmosphere. As shown in Fig. 3a, the diffraction peaks of the Ni_3_Zn (111) and (200) planes decreased gradually, whereas the intensity of Ni_3_ZnC_0.7_ (111) and (200) peaks increased until a complete transformation of Ni_3_Zn to Ni_3_ZnC_0.7_ occurred with a temperature preservation at 200 °C during 1 h. During the subsurface structure changed from hydrogen to carbon atoms, the selectivity towards ethylene increased highly from 30.6% to 94.5% (Fig. 3b), while the conversion of acetylene decreased from 99.2% to 56.3%. In general, the intermediate interstitial structure is metastable and depend kinetically on the chemical potential of desired elements^14^. Herein, the Ni_3_ZnC_0.7_/oCNT catalyst exhibits a superior and stable selectivity for a variety of H_2_/C_2_H_2_ ratios (Fig. 3c) due to the removal of the subsurface interstitial H hydrogenation pathway, which was evidenced by a series of H_2_ temperature-programmed desorption experiments of Ni/oCNT, Ni_3_Zn/oCNT, and Ni_3_ZnC_0.7_/oCNT catalysts (Supplementary Fig. 10). For a ratio in the range of 3–15, Ni_3_ZnC_0.7_/oCNT yields a 99.9–86.1% selectivity towards ethylene, and a 0.1–13.9% selectivity towards ethane in the conversion range of 9.7–93.5%. Generally, the reaction probabilities between adsorbed H and intermediate ethylene would increase as the coverage of activated H increases with the H_2_ concentration. Also, it is the highly exothermic feature of ethylene hydrogenation that leads further to the increase of ethane selectivity and uncontrollable temperature runaway^13,36^. Therefore, the high selectivity of the Ni_3_ZnC_0.7_ catalyst within a wide H_2_/C_2_H_2_ ratio range is industrially important, which allows for stable operation under fluctuated conditions. In addition, the selectivity control under a constant high conversion is challenging but desirable to eliminate the trace amount acetylene in ethylene. As shown in Fig. 3d, the obtained Ni_3_ZnC_0.7_/oCNT catalyst exhibits 94% selectivity toward ethylene under 99% conversion in a 720 min reaction, which is superior to Ni/oCNT catalyst (27% selectivity at the initial 99% conversion). The Ni/oCNT catalyst exhibits a rapidly reduced activity and poor stability under reaction conditions. This is supposed to the segregation and accumulation of carbonaceous fragments on the Ni surface and consistent with previous studies^17,37–39^. Obviously, the decreased conversion of alkyne for Ni catalyst cannot meet the industrial requirements and may poison the following polymerization catalysts. However, the Ni_3_ZnC_0.7_/oCNT catalyst exhibits excellent stability in comparison with Ni. That is, the introduction of carbon atoms not only inhibits the unselective hydrogenation pathway but also regulates the electronic structure of Ni. Therefore, it suppresses the further dissociation of adsorbed acetylene molecules. Thus, the stability of Ni_3_ZnC_0.7_/oCNT catalyst is significantly improved in comparison with Ni (Supplementary Fig. 11). The reaction carbon balance of Ni_3_ZnC_0.7_/oCNT catalyst was evaluated in the absence of ethylene. As shown in Supplementary Fig. 12, the Ni_3_ZnC_0.7_/oCNT catalyst exhibits good stability and improved selectivity compared with ethylene-rich condition during 13 h reaction. The carbon balance is almost 100% during the test. In addition, the noble metal-based Pd/oCNT and PdAg/oCNT catalysts synthesized via the same impregnation method (Supplementary Figs. 13 and 14) are also compared with the Ni_3_ZnC_0.7_/oCNT. Both the carbon-based support and the addition of Ag led to a selectivity enhancement in this reaction as previously reported^40,41^. Because of carbonaceous deposition during the reaction, the Pd (97–70%) and PdAg (93–57%) catalysts showed poor stability (Supplementary Figs. 15 and 16) compared with Ni_3_ZnC_0.7_^42–44^. The thermogravimetric analysis (TGA) experiments were conducted to evaluate the carbon deposition on the spent Ni_3_Zn/oCNT and Ni_3_ZnC_0.7_/oCNT catalysts. As shown in Supplementary Fig. 17, the fresh and spent Ni_3_ZnC_0.7_/oCNT catalysts exhibit a similar initial weight loss at 395 ^o^C, which is supposed to the combustion of oCNT. The weight loss occurs at 240 °C of the spent Ni_3_Zn/oCNT, however, which is much lower than that of fresh Ni_3_Zn/oCNT. It indicates the strongly adsorbed carbonaceous deposition on the surface of Ni_3_Zn after 10 h reaction at 100 °C. These TGA results further prove that the carbonaceous accumulation is highly suppressed on the surface of Ni_3_ZnC_0.7_/oCNT catalysts. These results indicate that the introduction of carbon atoms to the interstitial sites of Ni enables an efficient suppression of the over-hydrogenation reaction pathway and the carbonaceous accumulation on the surface (Supplementary Fig. 18). As a result, it affords the Ni_3_ZnC_0.7_/oCNT with high activity and selectivity, i.e., a stable catalyst in acetylene hydrogenation reaction. In addition, the selectivity control in acetylene hydrogenation is more challenging under high-pressure condition, which should be carefully taken into account^45,46^.

### Electronic properties of Ni with Zn and C atom inclusion

**Fig. 4:**
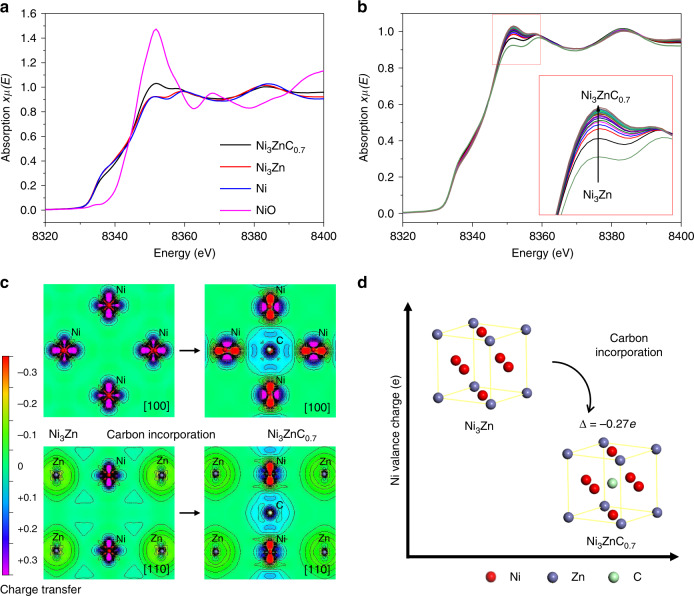
Electronic structure of Ni in Ni_3_Zn and Ni_3_ZnC_0.7_. **a** Normalized Ni K-edge XANES spectra of NiO (pink curve), Ni foil (blue curve), Ni_3_Zn (red curve), and Ni_3_ZnC_0.7_ (black curve), respectively. **b** In situ Ni K-edge XANES spectra during the process from Ni_3_Zn to Ni_3_ZnC_0.7_ under 1.0 vol% C_2_H_2_/He atmosphere at 200 °C. **c** Charge density difference plots of the interface along [100] and [110] directions of Ni_3_Zn and Ni_3_ZnC_0.7_. **d** The charge density variation of Ni when transformed from Ni_3_Zn to Ni_3_ZnC_0.7_. Δ represents that the introduction of C to Ni_3_Zn renders Ni with an electron deficiency of −0.27 per atom.

The variation of the electronic structure of Ni caused by the charge transfer between C and Ni in Ni_3_ZnC_0.7_ is demonstrated in Fig. 4. After calibration with reference samples (Ni foil and Ni oxide), the X-ray absorption near edge structure (XANES) spectra of the Ni K-edge were conducted for the Ni_3_Zn/oCNT and Ni_3_ZnC_0.7_/oCNT catalysts. It clearly shows that Ni is indeed electron-deficient in Ni_3_ZnC_0.7_ compared with Ni and Ni_3_Zn (Fig. 4a). Furthermore, in situ XANES experiments reveal the evolution of the electronic structure of Ni from Ni_3_Zn to Ni_3_ZnC_0.7_. The intensity of the white line (Fig. 4b) increases from Ni_3_Zn gradually to Ni_3_ZnC_0.7_, which suggests Ni electron deficiency with the incorporation of carbon. The charge transfer distribution maps (Fig. 4c) acquired from the density functional theory (DFT) calculations^47^ was applied. It clearly displays the charge density differences in Ni_3_Zn and Ni_3_ZnC_0.7_ along the [100] and [110] directions, respectively. In Ni_3_Zn, a charge transfer occurs from Zn to Ni because of the electronegativity differences between Ni (1.88) and Zn (1.66). As a result, Ni has an electron richness of 0.138 per atom. After C incorporation, an obvious charge transfer occurs from Ni to C, rendering Ni with an electron deficiency of −0.133 per atom (Fig. 4d). Since the interaction is negligible between Zn and C (Fig. 4c), there is no charge transfer in this case. Therefore, the positive charge and the lower d-band center (Supplementary Fig. 19) of Ni in Ni_3_ZnC_0.7_ suppresses the dissociation of acetylene and weakens the adsorption of acetylene/ethylene, which contributes to the decreased polymerization products. In short, it leads to improved stability for highly efficient acetylene hydrogenation.

## Discussion

This work demonstrates that highly efficient hydrogenation of alkyne could be obtained via introducing carbon atoms within Ni lattice containing an expanded interstitial space. With an addition of 25% Zn, the interstitial space of Ni is expanded with a well-maintained capability to adsorb and dissociate hydrocarbon molecules. Thus, the dissociated carbon atoms enable readily penetrating into Ni_3_Zn to offer the formation of Ni_3_ZnC_0.7_ structure under acetylene contained atmosphere at 200 °C. Both XRD and TEM investigations confirm the lattice parameter expansion after the inclusion of Zn and carbon. EDX and EELS analysis explicitly reveal the existence of carbon in Ni_3_ZnC_0.7_. Combined with in situ XANES experiments and DFT calculations, the introduced carbon atoms coordinate with the neighboring Ni atoms and impair a positive charge to Ni. As a result, the interstitial occupation in Ni_3_ZnC_0.7_ suppresses the reaction pathway of ethylene hydrogenation and increases the selectivity drastically under hydrogen-rich/poor conditions. That is to say, it can effectively bypass the temporal metastable state variation under a fluctuation reaction condition. More importantly, the formed Ni_3_ZnC_0.7_ exhibits a highly improved stability compared with Ni. As a result, the dissociation and accumulation of carbonaceous species are inhibited on the surface. Hence, our work provides a proof-of-concept to control of interstitial sites in heterogeneous metal catalyst. Moreover, such a new solution enables improving the selectivity and stability for selective acetylene hydrogenation.

## Methods

### Catalysts preparation

The multiwall CNTs was purchased from Shandong Dazhan Nano Materials Co., Ltd, China. The CNTs were firstly treated with HCl and then oxidized with concentrated HNO_3_ at 140 °C for 2 h (oCNT). The Ni_3_Zn/oCNT catalyst was synthesized with a typical impregnation method. For the 5.0 wt% Ni_3_Zn/oCNT, 101 mg Ni(NO_3_)_2_ and 33.7 mg Zn(NO_3_)_2_ were dissolved in 40 mL ethanol, then 374 mg oCNT was added into the solution and the suspension was ultrasonic dispersed for 1 h to obtain a homogeneous distribution. The ethanol was evaporated at 50 °C using a rotary evaporator. After calcination at 100 °C for 2 h in static air, the reduction process was conducted at elevated temperature with a heating rate of 10 °C min^−1^ from 30 to 500 °C for 2 h in 50.0 vol% H_2_ in Ar with the flow rate of 100 mL min^−1^. The Ni_3_Zn/oCNT catalyst was obtained after cooling to room temperature. The Ni_3_ZnC_0.7_/oCNT catalyst prepared by switching the gas atmosphere to 1.0 vol% C_2_H_2_/He at 200 °C for 1 h after the reduction of Ni_3_Zn/oCNT. The 5.0 wt% Ni/oCNT, 2.0 wt% Pd/oCNT and PdAg/oCNT catalysts were also synthesized with the same impregnation method and reduced at 500 °C.

### XRD investigations

The in situ XRD measurements were conducted on a PANalytical X’pert PRO diffractometer with XRK900 in situ chamber using CuKα radiation, operated at 40 kV and 30 mA. The 10.0 wt% loading of Ni and Zn on oCNT was chosen for a clear peak evolution considering that 5.0 wt% loading sample would cause low intensity of the diffraction peaks and lead to ambiguity in peak recognition in the in situ XRD experiments. The 10.0 wt% Ni_3_Zn/oCNT sample was transferred to an in situ chamber with the beryllium window. Then the H_2_/He flow at 10 mL min^−1^ was introduced after the He flushing, the temperature elevated with a rate of 10 °C min^−1^ from room temperature to 500 °C while the flow is steady. The XRD patterns were recorded at desired temperatures from 10° to 90°. After the reduction, the chamber was cooled down to room temperature and flushed with helium. Then the acetylene was imported and the evolution of the Ni_3_Zn structure to Ni_3_ZnC structure was examined at elevated temperature to 200 °C under 1.0 vol% C_2_H_2_/He flow (10 mL min^−1^). The ex situ XRD patterns were performed on a Rigaku D/max 2400 diffractometer (CuKα radiation, *λ* = 0.15418 nm).

### X-ray absorption spectroscopy investigation

The Ni K-edge XANES experiments were performed at the beamline 1W1B of the Beijing Synchrotron Radiation Facility, Institute of High Energy Physics, Chinese Academy of Sciences. The electron storage ring was operated at 2.5 GeV. Using a Si(111) double-crystal monochromator; all the data collections were carried out in the transmission mode for the Ni K-edge. A Ni foil was measured simultaneously and used for the energy calibration. The XAS data analysis was carried out with the programs of IFEFFIT. In situ Ni K-edge XANES spectra of Ni_3_Zn/oCNT catalyst were carried out in an in situ reaction cell. The powdered sample was pressed into sheet and loaded into a reactor cell equipped with polyimide windows. Then, the sample was reduced in 50.0 vol% H_2_/He at 500 °C for 1 h at a heating rate of 10 °C min^−1^, and subsequently lower down to 200 °C and flushed with He for 30 min. Then 1 vol% C_2_H_2_/He atmosphere was switched into the in situ reaction cell, and the Ni K-edge XANES spectra were collected within 30 min.

### H_2_ temperature-programmed desorption

H_2_-TPD experiments combined with on-line mass spectroscopy were conducted to evaluate the hydrogen adsorption/absorption strength of Ni/oCNT, Ni_3_Zn/oCNT, and Ni_3_ZnC_0.7_/oCNT catalysts. Fifty milligrams of samples were pre-treated with a 10.0 vol% H_2_/He mixture (40 mL min^−1^) at 500 °C for 1 h, switched to He (40 mL min^−1^) to flush for 30 min after lowered down to room temperature. The Ni_3_ZnC_0.7_ catalyst was in situ prepared after hydrogen reduction. The mass signal of *Mz* = 2 was recorded at a heating rate of 10 °C min^−1^ to 500 °C for three samples.

### Thermogravimetric analysis

TGA experiments were conducted by using a Netzsch-STA 449 F3 instrument. Fresh and spent catalysts (~1 mg) were treated in 50% O_2_/Ar flow (40 mL min^−1^) from 40 to 700 °C with a heating rate of 5 °C min^−1^.

### TEM investigations

Ni_3_Zn/oCNT and Ni_3_ZnC_0.7_/oCNT samples were dispersed and ultrasoniced in ethanol for 10 min, and then a drop of the solution was placed on a holey C/Cu TEM grid to be used for the TEM characterization. An FEI Tecnai G^2^ F20 microscope, JEM ARM200F and 300F Grand ARM Cs-corrected microscopes equipped with EDX and HAADF detectors were used to perform microstructural investigations of a series of Ni_3_Zn/oCNT and Ni_3_ZnC_0.7_/oCNT samples in both TEM and STEM modes.

### Temperature-programmed surface reaction

TPSR of C_2_H_2_ on the Ni/oCNT and Ni_3_Zn/oCNT sample were conducted with a quartz tubular micro-reactor connected to an on-line mass spectrometer. Fifty milligram samples were pre-treated with a 10.0 vol% H_2_/He mixture (40 mL min^−1^) at 500 °C or 1 h, switched to He (40 mL min^−1^) to flush for 30 min after lower down to room temperature, then switched to 1.0 vol% C_2_H_2_/He atmosphere (40 mL min^−1^). The mass signal of *Mz* = 2 was recorded at a heating rate of 4 and 10 °C min^−1^ to 200 °C for Ni and Ni_3_Zn samples, respectively.

### Theoretical calculation

The DFT calculations were performed with Generalized Gradient Approximation using the Perdew–Burke–Ernzerhof (PBE) exchange-correlation function. By using WIEN2k code, the charge density difference was calculated with a plane-wave cutoff parameter of RK_max_ = 7 and 1000 points in the whole Brillouin zone. The bader charge analysis and geometric relaxation of Ni_3_Zn was implemented using the Vienna ab-initio simulation package (VASP). The kinetic energy cutoff of the plane-wave was set to be 400 eV, and the Brillouin zone was sampled by Monkhorst–Pack meshes of 9 × 9 × 9 grid. Atomic position and lattice parameters of Ni_3_Zn were geometrically relaxed until changes in the total energy and force were less than 10^−4^ eV and 5 × 10^−2^ eV Å^−1^, respectively.

### Catalytic tests

The acetylene selective hydrogenation in the excess of ethylene (1.5, 3.0, 4.5, 6.0, and 7.5 vol% H_2_, 20.0 vol% C_2_H_4_, 0.5 vol% C_2_H_2_, helium as balance) was performed in a fixed-bed quartz micro-reactor under atmospheric pressure. The Ni_3_Zn/oCNT was in situ reduced and transformed to Ni_3_ZnC_0.7_/oCNT at 200 °C under reaction conditions. For the stability test, the Ni_3_ZnC_0.7_/oCNT sample was in situ generated after reduction and carburization processes following 20 mL min^−1^ 50 vol% H_2_/He treatment at 500 °C for 2 h and 200 °C 1.0 vol% C_2_H_2_/He for 1 h before the reaction. Ni/oCNT catalyst was also in situ reduced at 500 °C for 2 h under 50 vol% H_2_/He atmosphere before the test.

The conversion (Conv) and selectively (Sele) were calculated as follows:

$${\mathrm{Conv}}_{{\mathrm{C}}_{\mathrm{2}}{\mathrm{H}}_{\mathrm{2}}}{\mathrm{ = }}\frac{{{\mathrm{C}}_{{\mathrm{C}}_{\mathrm{2}}{\mathrm{H}}_{\mathrm{2}}{\mathrm{,in}}} - {\mathrm{C}}_{{\mathrm{C}}_{\mathrm{2}}{\mathrm{H}}_{\mathrm{2}}{\mathrm{,out}}}}}{{{\mathrm{C}}_{{\mathrm{C}}_{\mathrm{2}}{\mathrm{H}}_{\mathrm{2}}{\mathrm{,in}}}}} \times {\mathrm{100\% }},$$

$${\mathrm{Sele}}_{{\mathrm{C}}_{\mathrm{2}}{\mathrm{H}}_{\mathrm{4}}}{\mathrm{ = }}\left( {{\mathrm{1}} - \frac{{{\mathrm{C}}_{{\mathrm{C}}_{\mathrm{2}}{\mathrm{H}}_{\mathrm{6}}{\mathrm{,out}}}}}{{{\mathrm{C}}_{{\mathrm{C}}_{\mathrm{2}}{\mathrm{H}}_{\mathrm{2}}{\mathrm{,in}}} - {\mathrm{C}}_{{\mathrm{C}}_{\mathrm{2}}{\mathrm{H}}_{\mathrm{2}}{\mathrm{,out}}}}}} \right) \times {\mathrm{100\% }},$$

where C_in_ represents the acetylene concentration in the feed gas and C_out_ represents the different products in the outlet gas. Because of high concentration of the ethylene and the minor changes caused by the hydrogenation of acetylene was not easy to be detected, the consumption of the acetylene was assumed to be either hydrogenated to ethylene or hydrogenated to ethane.

## Supplementary information

Supplementary Information

## Data Availability

More experimental details and additional data can be found in the Supplementary Information (Supplementary Figs. 1–19 and Notes 1–4). All the relevant data are available from the corresponding author upon reasonable request. Source data are provided with this paper.

## Source data

Source Data
