# Supplementary material for: Manipulating interstitial carbon atoms in the nickel octahedral site for highly efficient hydrogenation of alkyne
Source: Nat Commun. 2020 Jul 3;11:3324. doi: 10.1038/s41467-020-17188-3 (PMC7335178; doi:10.1038/s41467-020-17188-3)
Supplement: Supplementary file 1 — Supplementary Information [file 41467_2020_17188_MOESM1_ESM.pdf]

Supplementary Information

Manipulating interstitial carbon atoms in the nickel octahedral site for highly efficient hydrogenation of alkyne

Niu et al.

Supplementary Figures

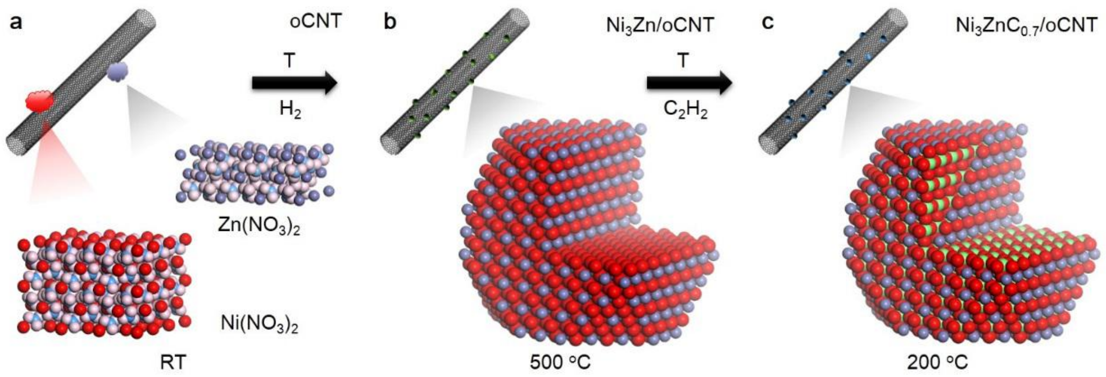

Supplementary Figure 1. Schematic diagram of preparation process.

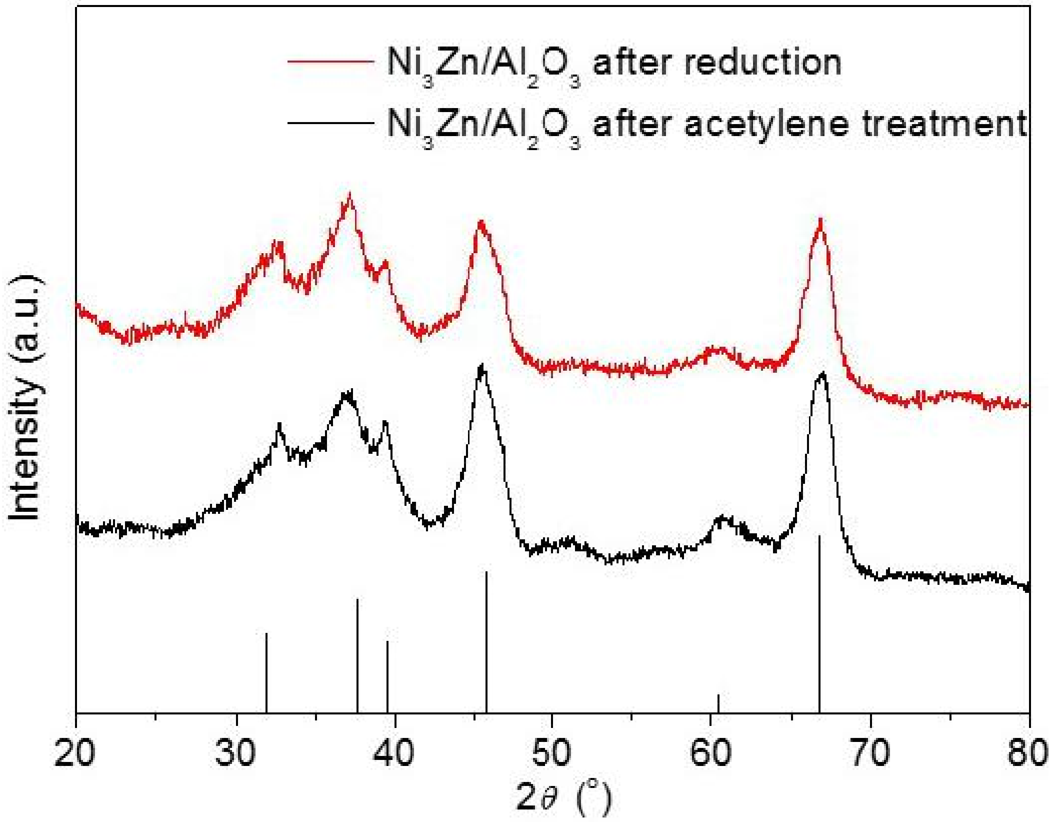

Supplementary Figure 2. XRD patterns of Al_2O_3 supported Ni/Zn NPs after 500 °C hydrogen and 200 °C acetylene treatment. The black diffraction lines are indexed as Al_2O_3 (JCPDS No. 29-0063).

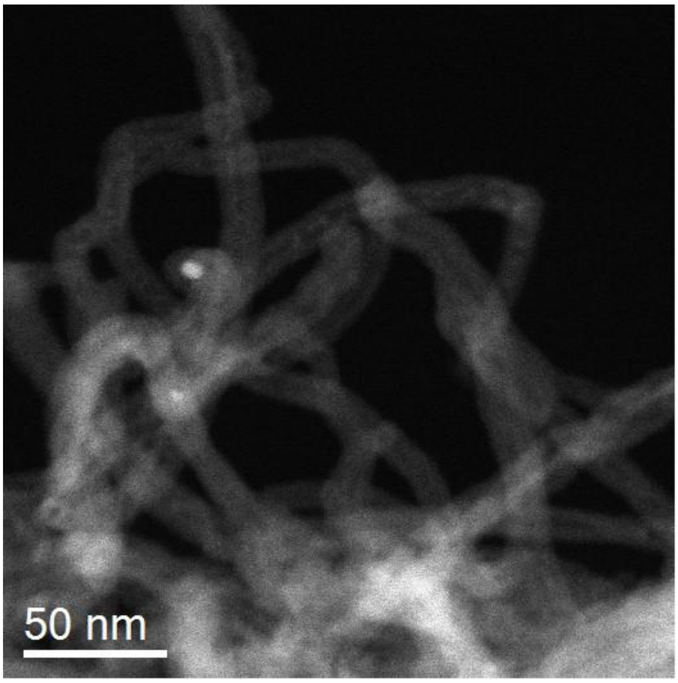

Supplementary Figure 3. STEM image of oCNT after impregnation with Ni and Zn salts.

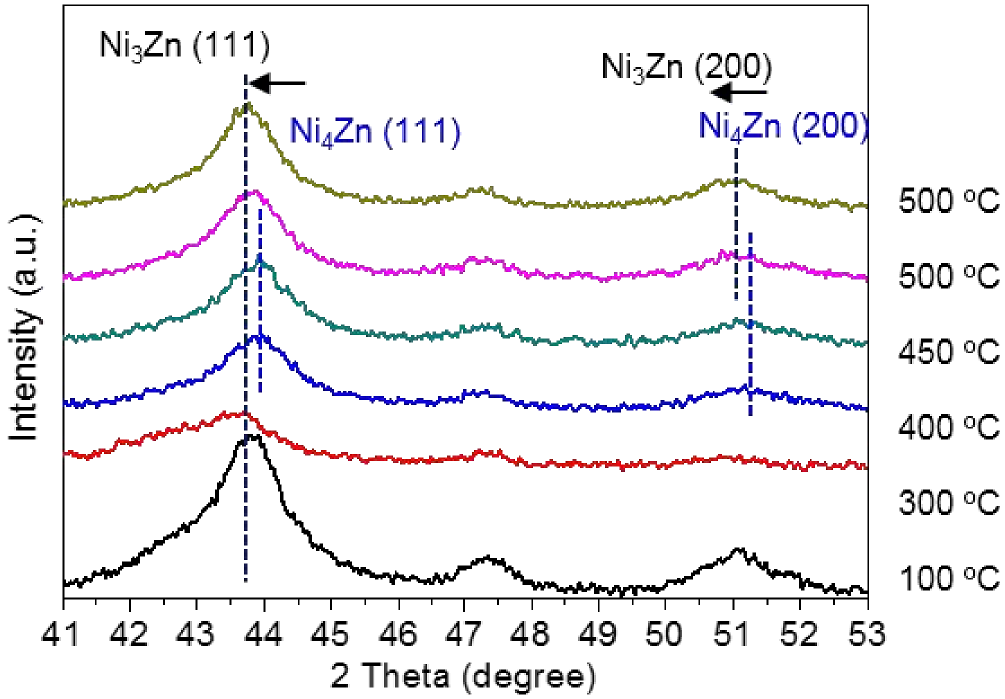

Supplementary Figure 4. In situ XRD patterns of the structural evolution of Ni_3Zn/oCNT under a 10.0 vol.% H_2/He atmosphere.

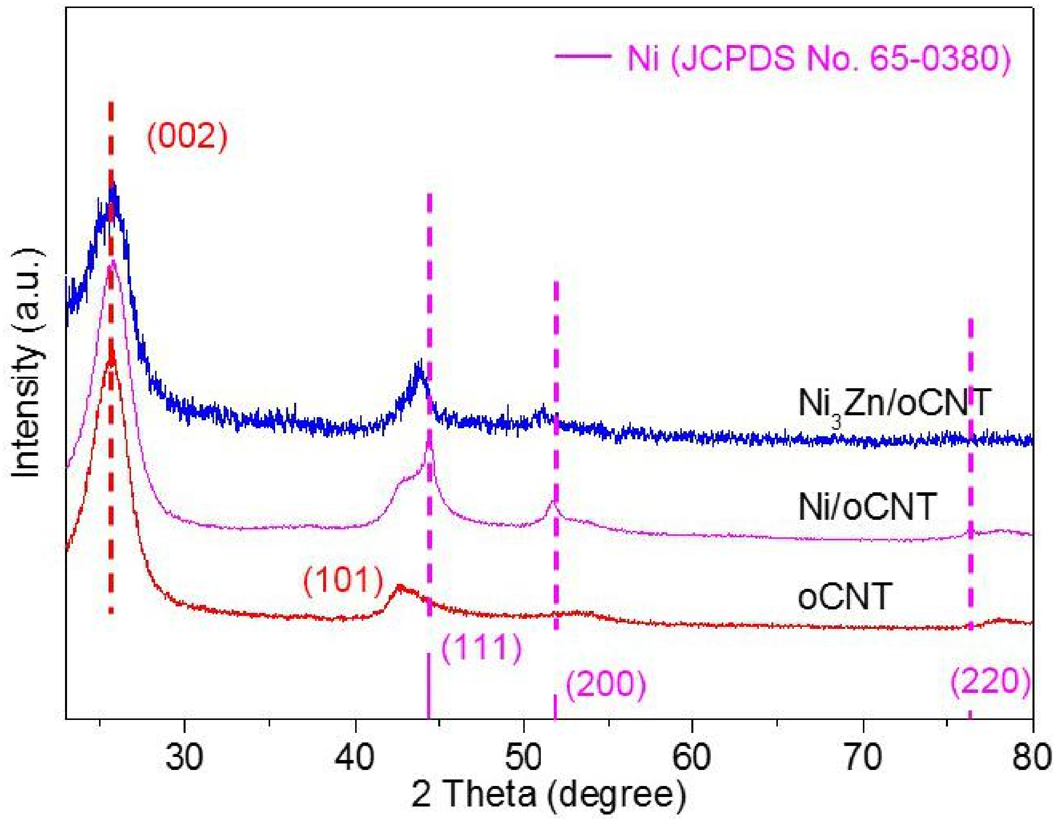

Supplementary Figure 5. XRD patterns of oCNT, Ni/oCNT and Ni_3Zn/oCNT after reduction for 2 h at 500 ^{o}C. The magenta diffraction lines are indexed as Ni (JCPDS No. 65-0380).

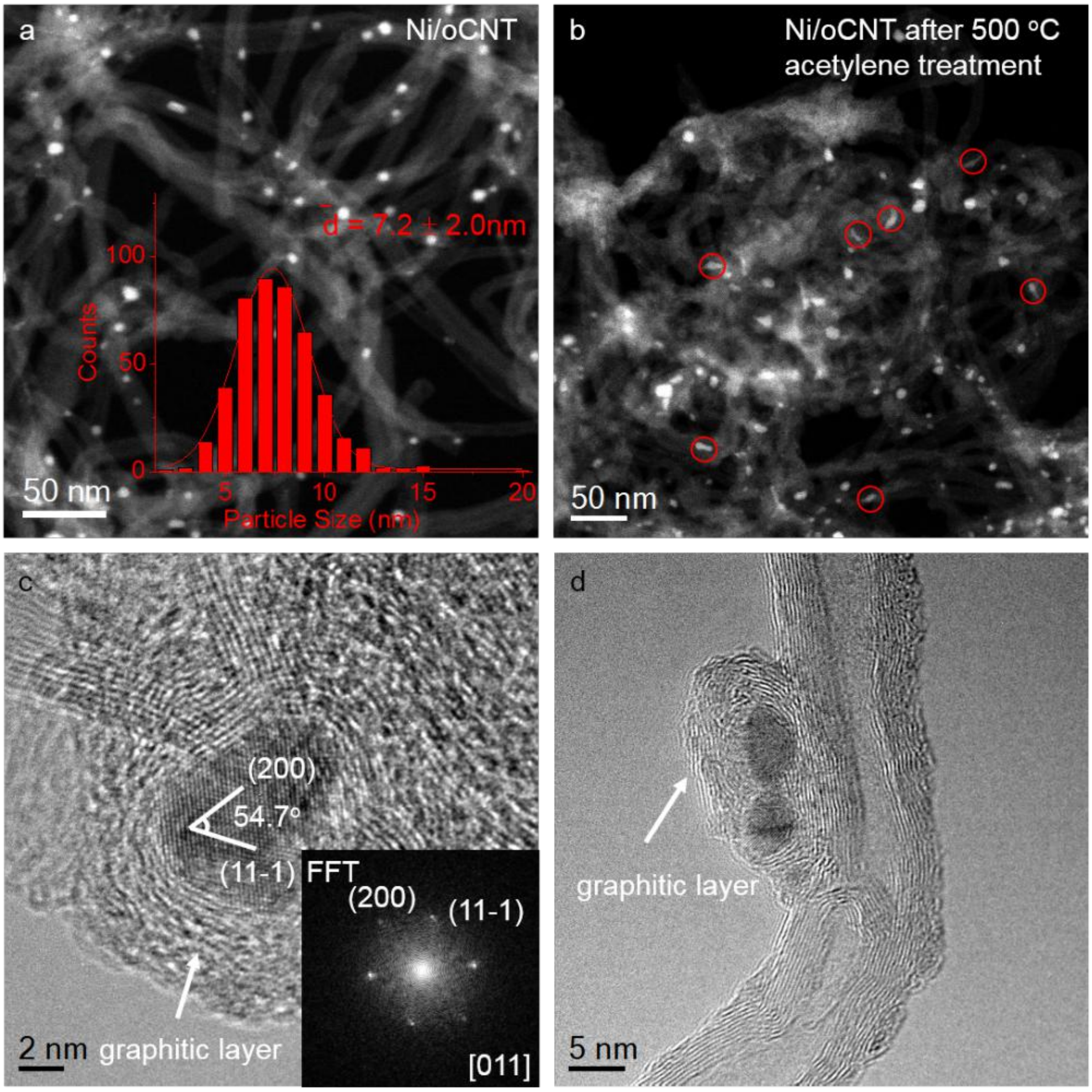

Supplementary Figure 6. HAADF-STEM image and particle size distribution (PSD) of Ni/oCNT (a) and after 500 ºC acetylene treatment (b). HRTEM images of the encapsulated Ni NPs (c, d), the right-down inset in c is the corresponding FFT.

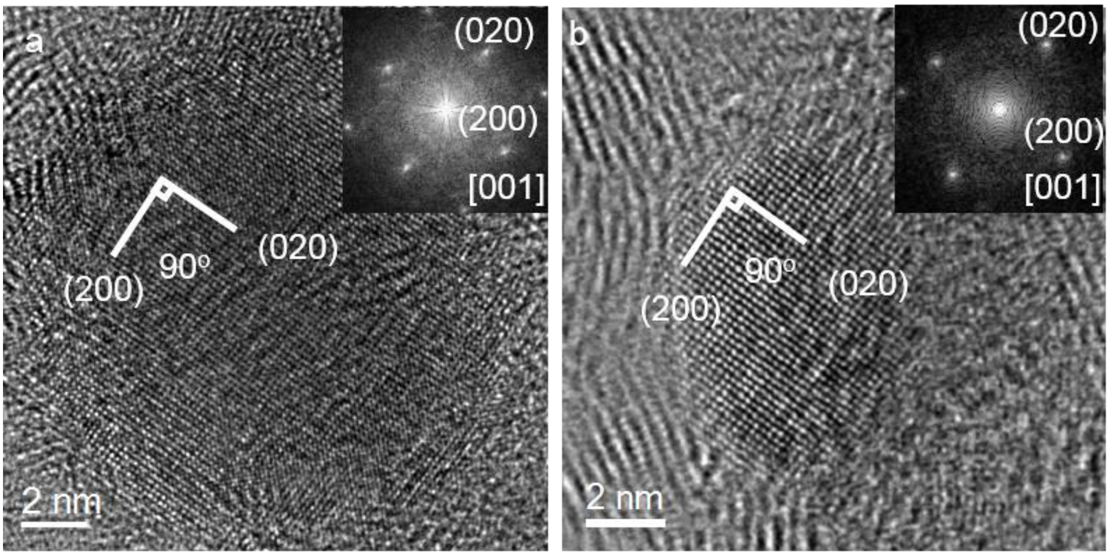

Supplementary Figure 7. HRTEM images aquired from [001] zone axis for Ni_3Zn/oCNT (a) and $Ni_3ZnC_{0.7}$/oCNT (b) catalysts, respectively. The insets in (a) and (b) are the corresponding FFTs.

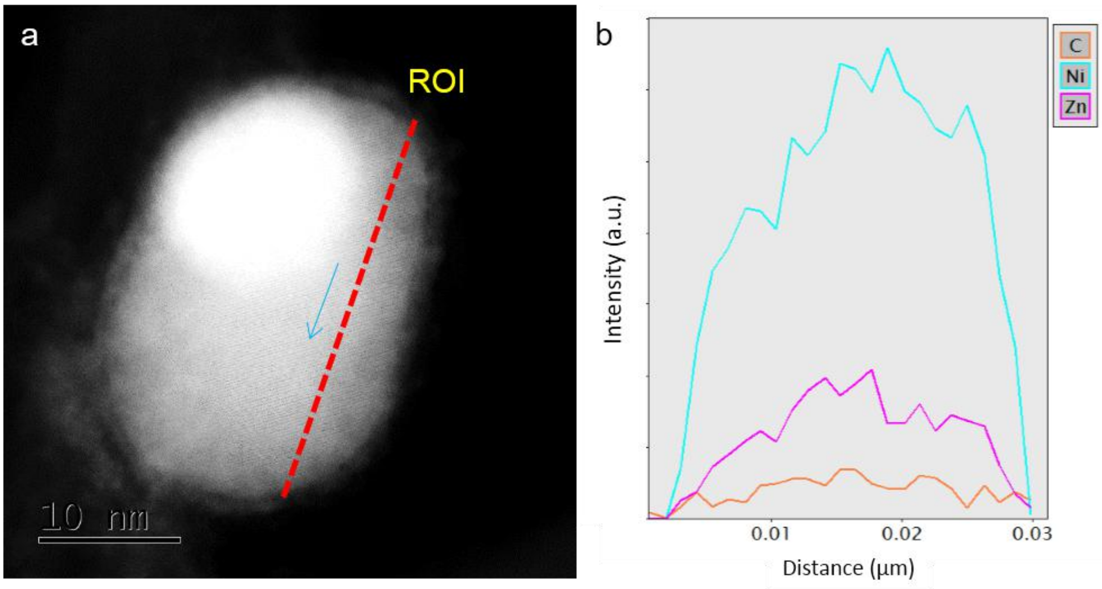

Supplementary Figure 8. STEM image (a) and corresponding EDX line profiles (b) of $Ni_3ZnC_{0.7}$ structure, which clearly reveal a uniform distribution and stoichiometric ratio of C, Ni and Zn across the nanoparticle.

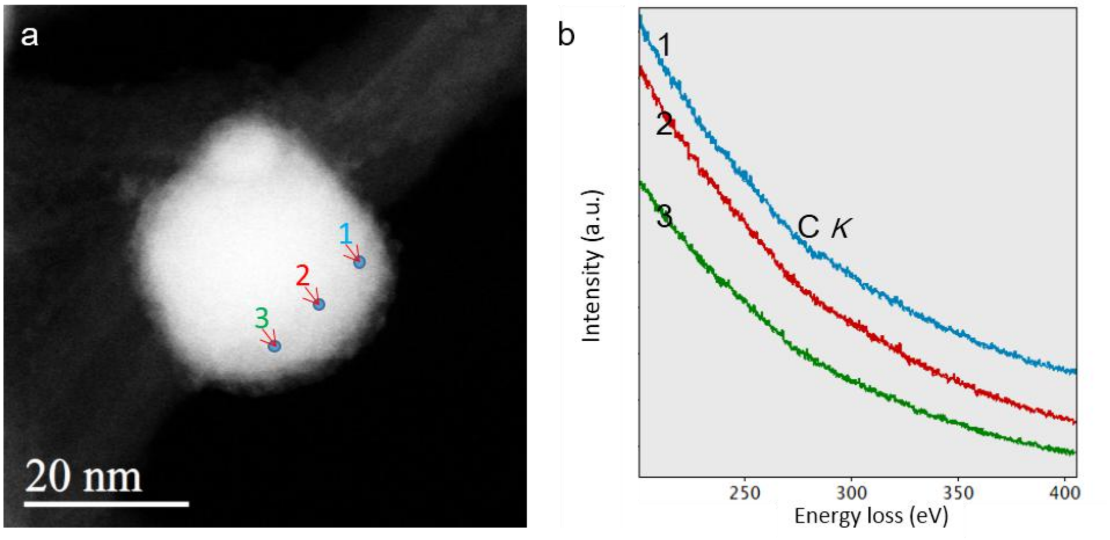

Supplementary Figure 9. STEM image (a) of Ni_3Zn/oCNT and the corresponding EEL spectra (b) recorded from different spots of the nanoparticle barely show carbon signal. The weak carbon *K* edge shown in the 1st spectrum is considerably due to the surface carbon species as a result of carbon contamination.

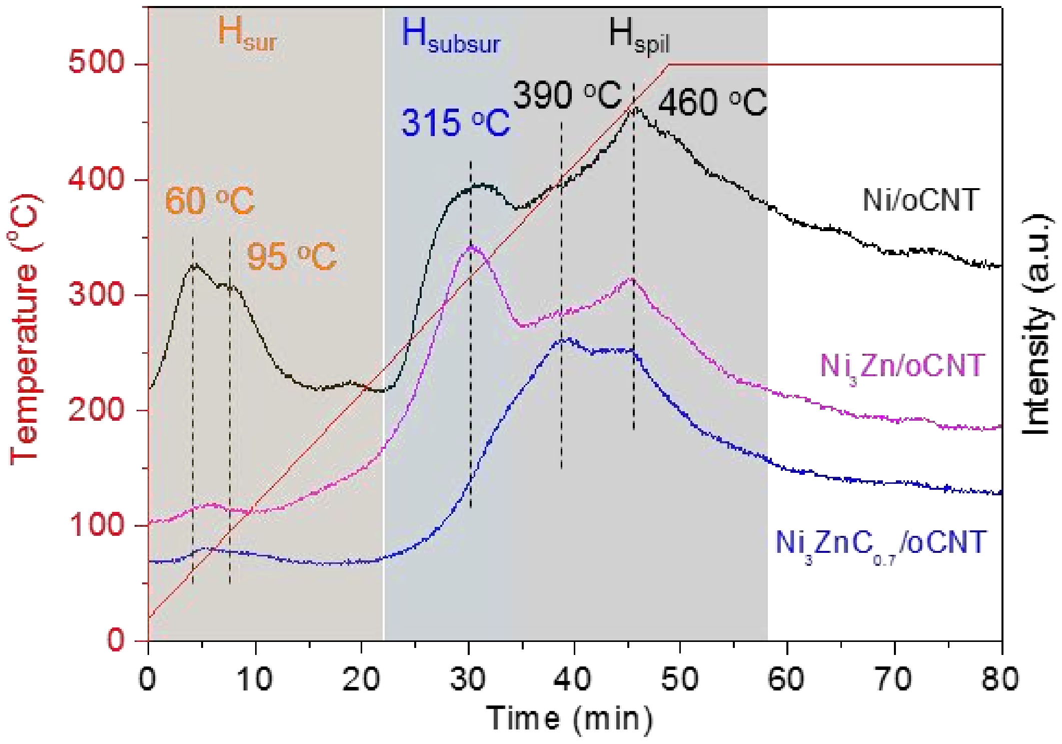

Supplementary Figure 10. H_2-TPD-MS results of Ni/oCNT, Ni_3Zn/oCNT, and $Ni_3ZnC_{0.7}$/oCNT catalysts at elevated temperatures under He atmosphere.

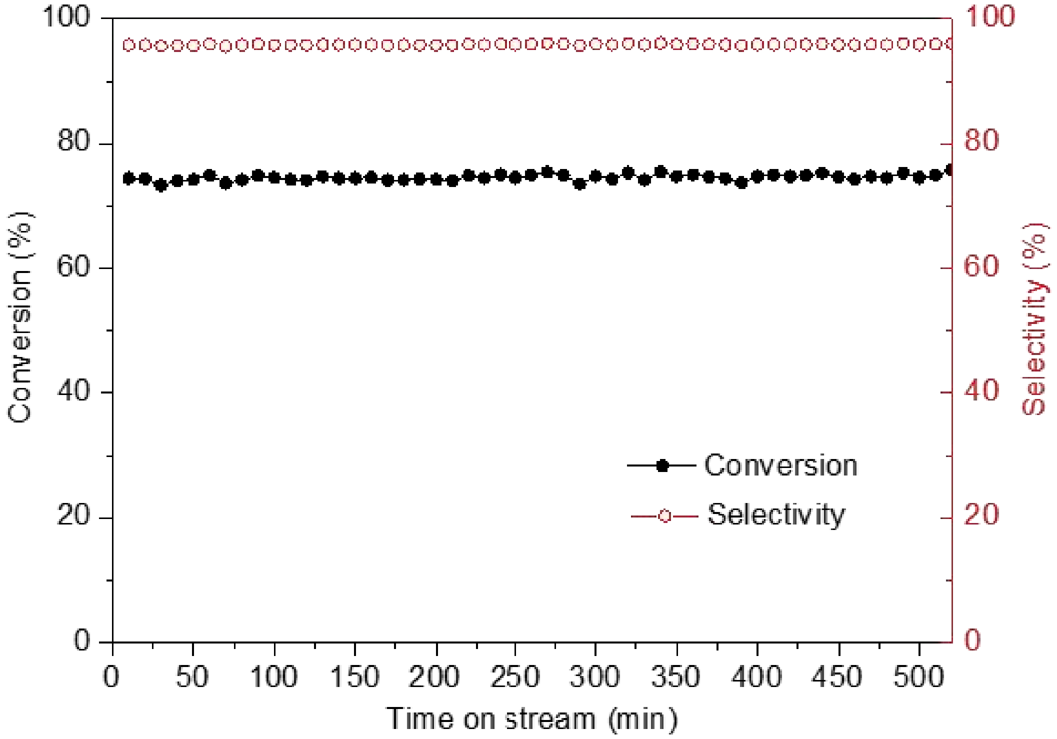

Supplementary Figure 11. Stability test of $Ni_3ZnC_{0.7}$/oCNT catalyst at low acetylene conversion. The solid and empty symbols represent the acetylene conversion and the selectivity towards ethylene. Reaction temperature: 200 °C; Reaction conditions: 4.5 vol.% H_2, 20 vol.% C_2H_4, 0.5 vol.% C_2H_2, helium as balance, GHSV = 360,000 ml g_{cat}^{-1} h^{-1}.

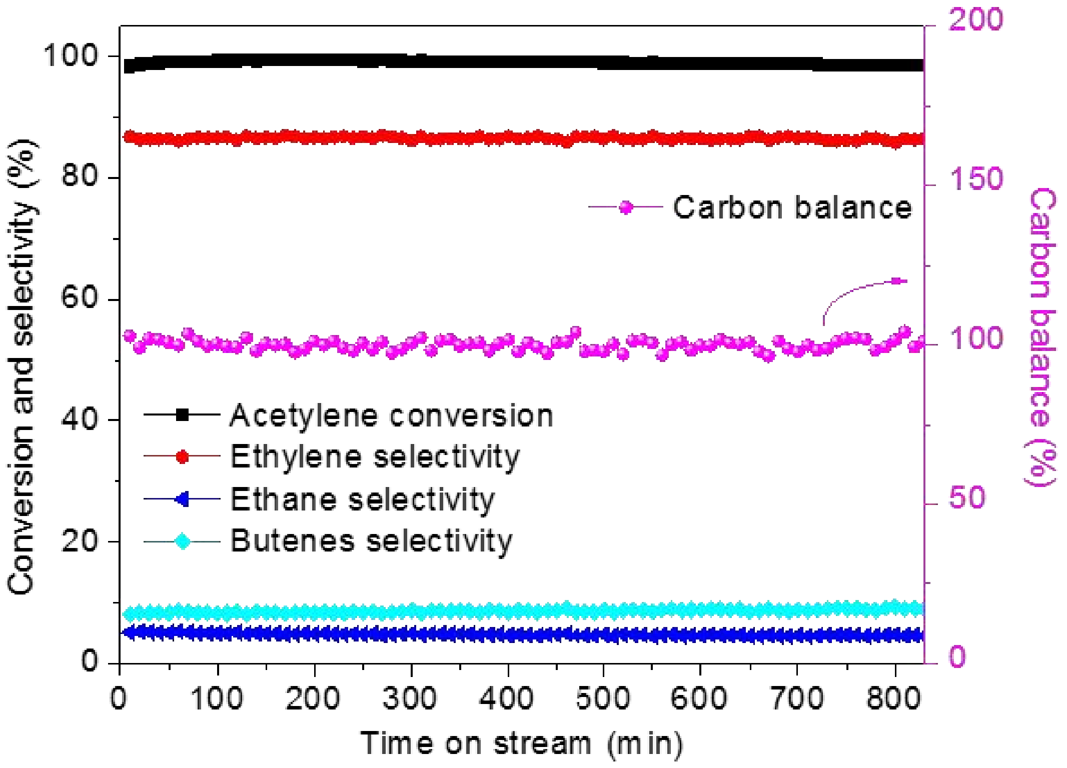

Supplementary Figure 12. Stability test of $Ni_3ZnC_{0.7}$/oCNT catalyst in the absence of ethylene. Reaction temperature: 200 °C; Reaction conditions: 9.0 vol.% H_2, 1.0 vol.% C_2H_2, helium as balance, GHSV = 60,000 ml g_{cat}^{-1} h^{-1}.

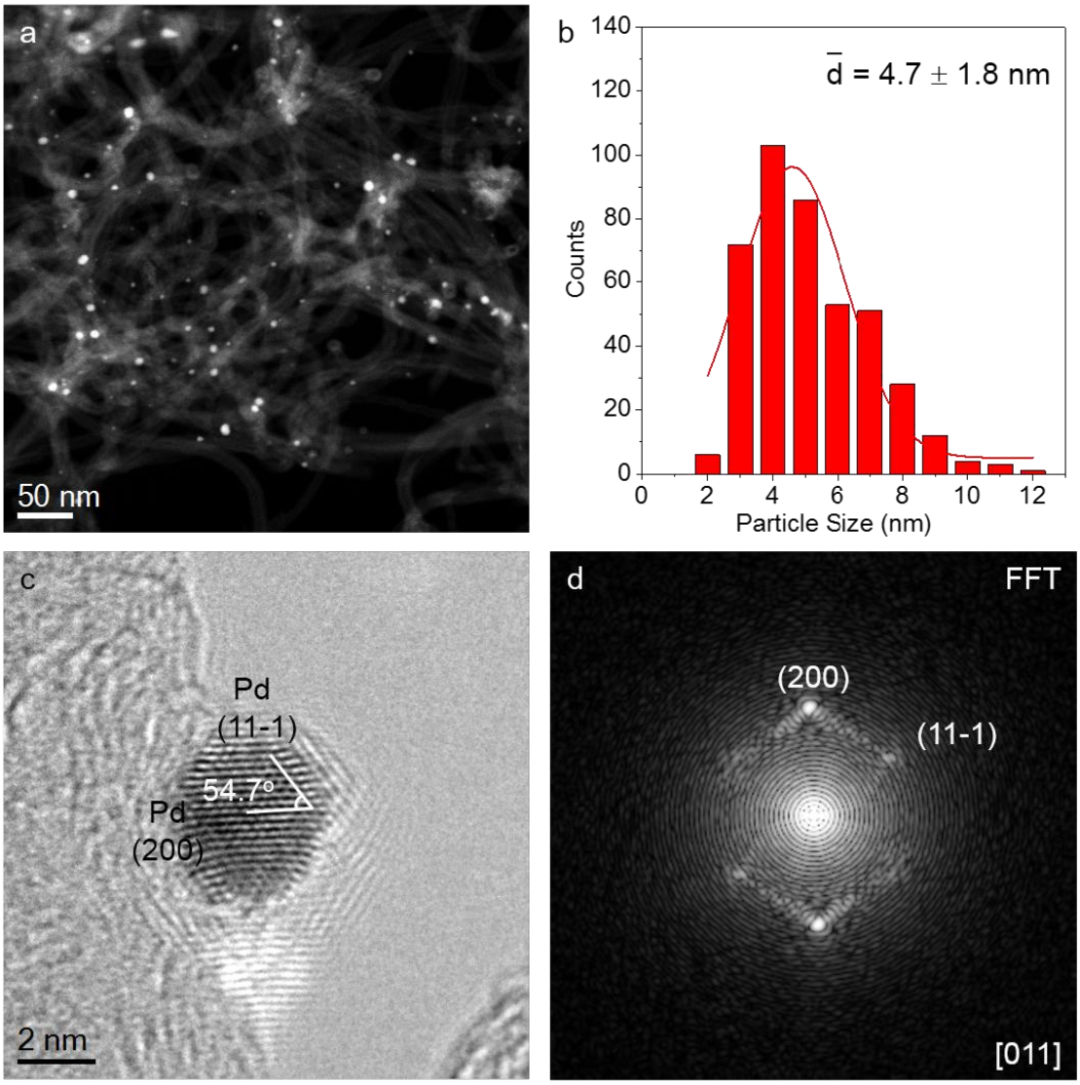

Supplementary Figure 13. STEM image (a) of Pd/oCNT sample after reduction at 500 °C. The corresponding PSD historgram (b) shows that the size of Pd NPs ranges from 2.0 to 12.0 nm with the average diameter is ~4.7 ± 1.8 nm. HRTEM image (c) of Pd/oCNT and the corresponding FFT (d).

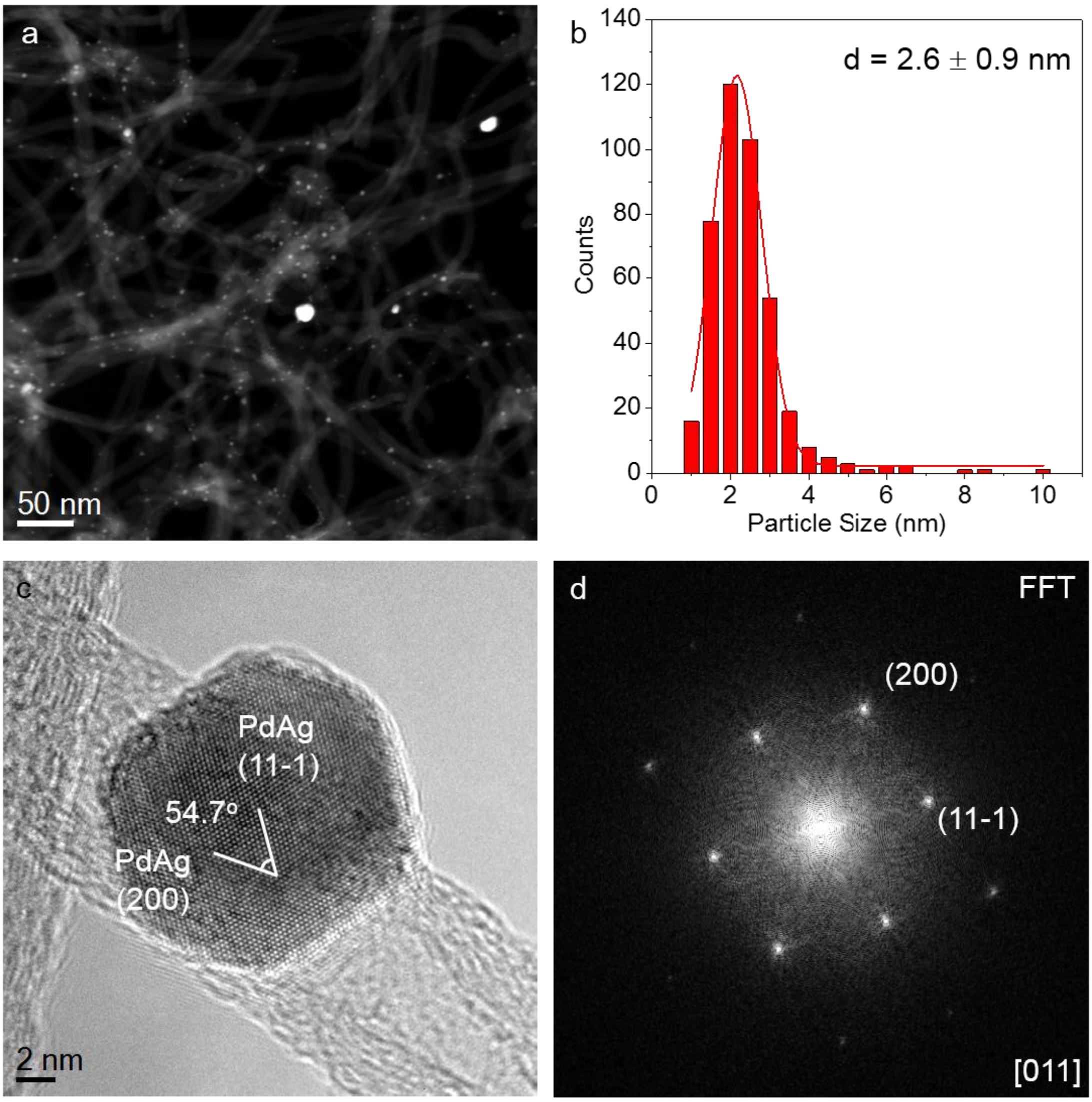

Supplementary Figure 14. STEM image (a) of PdAg/oCNT sample after reduction at 500 °C. The corresponding PSD historgram (b) shows that the size of PdAg NPs ranges from 1.0 to 10.0 nm with the average diameter is ~2.6 ± 0.9 nm. HRTEM image (c) of PdAg/oCNT and the corresponding FFT (d).

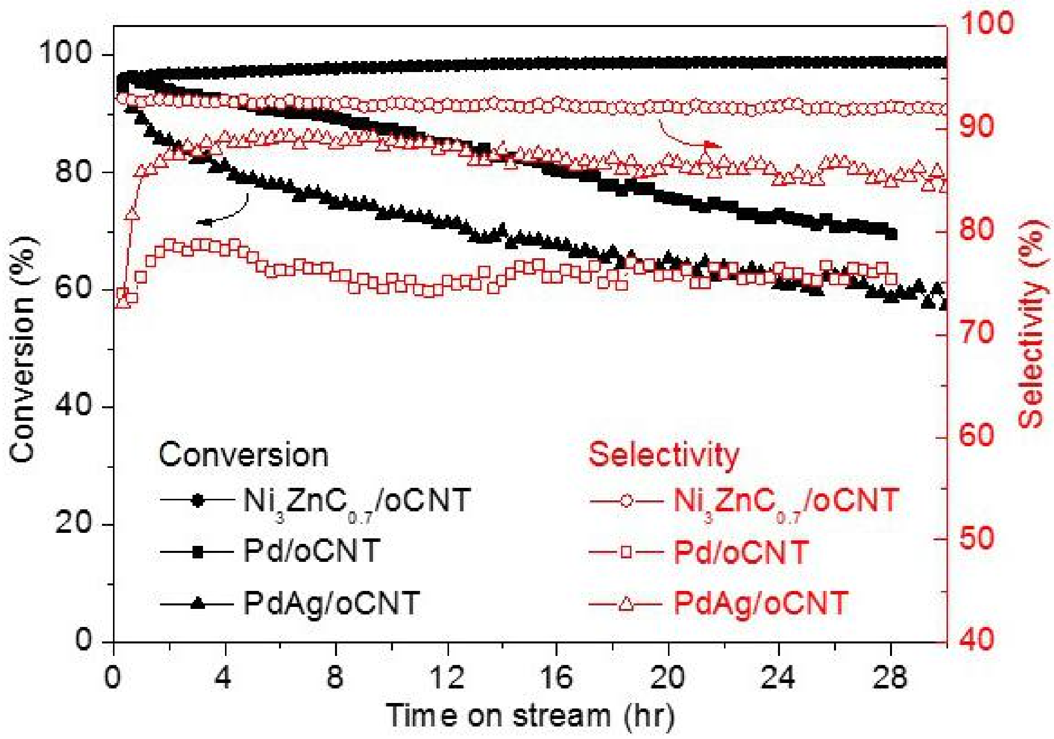

Supplementary Figure 15. Stability test of Pd/oCNT (0.02 mg), PdAg/oCNT (0.02 mg) and $Ni_3ZnC_{0.7}$/oCNT (10.0 mg) catalysts. The solid and empty symbols represent the acetylene conversion and the selectivity towards ethylene. Reaction temperature: 200 °C; Reaction conditions: 4.5 vol.% H_2, 20 vol.% C_2H_4, 0.5 vol.% C_2H_2, helium as balance, flow rate = 40 mL min^{-1} for $Ni_3ZnC_{0.7}$/oCNT, 20 mL min^{-1} for Pd/oCNT and PdAg/oCNT.

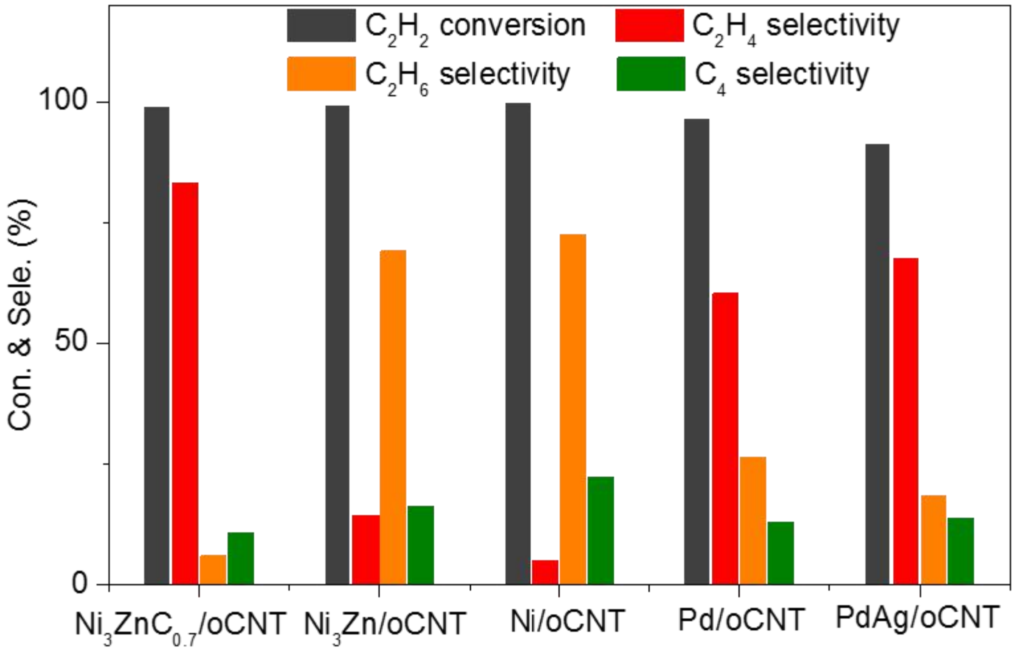

Supplementary Figure 16. Acetylene conversion, ethylene, ethane and butenes selectivity of $Ni_3ZnC_{0.7}$/oCNT, Ni_3Zn/oCNT, Ni/oCNT, Pd/oCNT and PdAg/oCNT samples. Reaction temperature: 200 ^{o}C; Reaction conditions: 4.5 vol.% H_2, 20 vol.% C_2H_4, 0.5 vol.% C_2H_2, hellium as balance, flow rate = 40 mL min^{-1}.

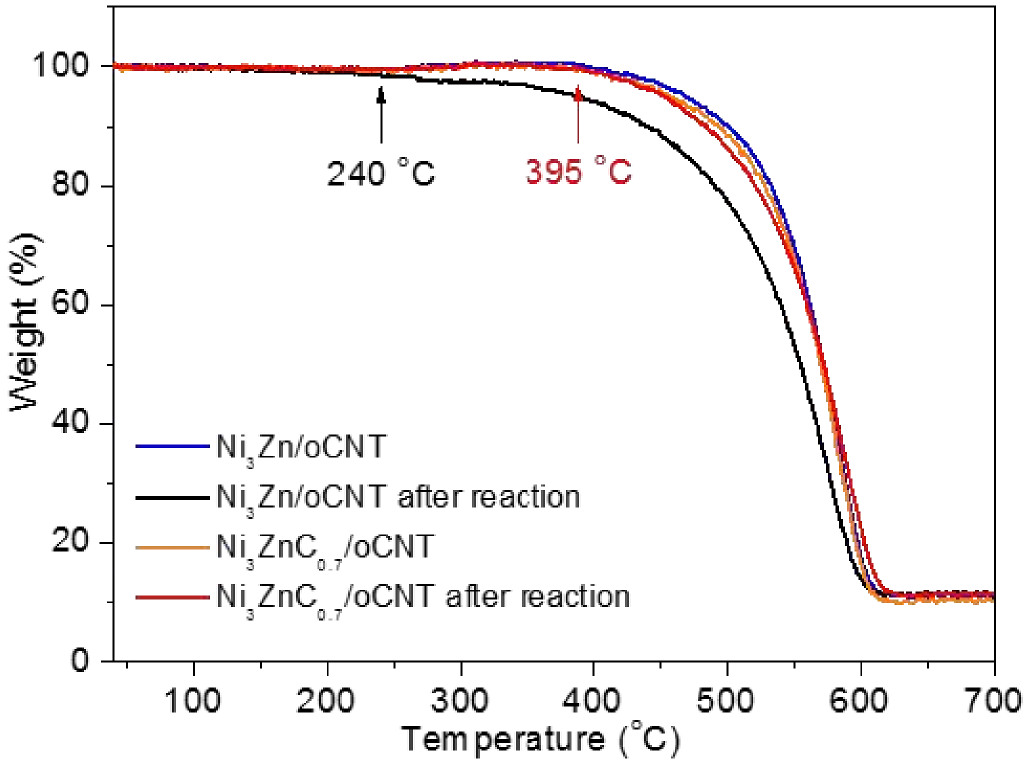

Supplementary Figure 17. TGA results of Ni_3Zn/oCNT and $Ni_3ZnC_{0.7}$/oCNT catalysts before and after reaction as a function of temperature under 50% O_2/Ar atmosphere.

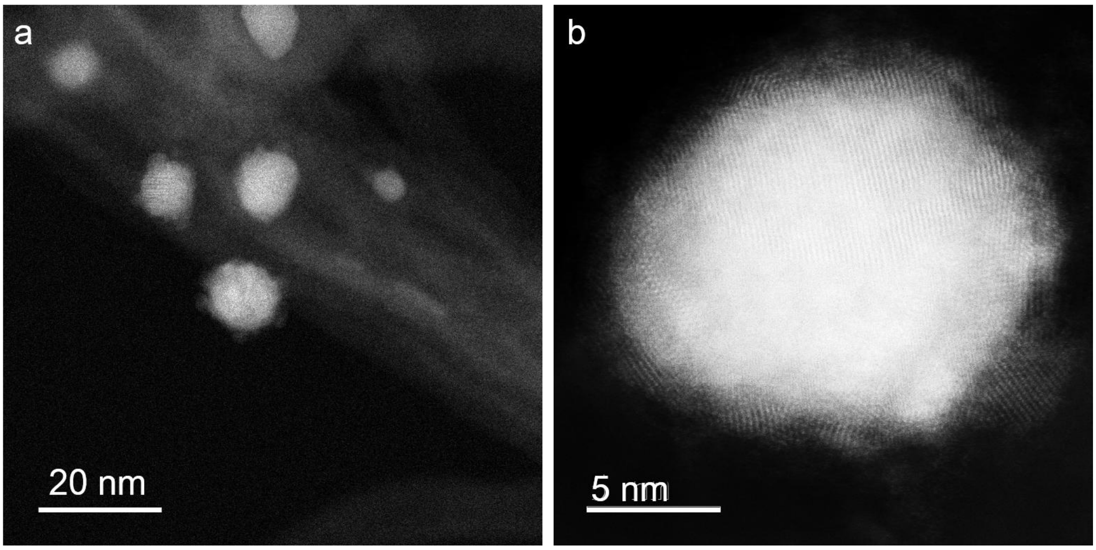

Supplementary Figure 18. Typical (a) and high resolution (b) STEM images of $Ni_3ZnC_{0.7}$/oCNT sample after reaction.

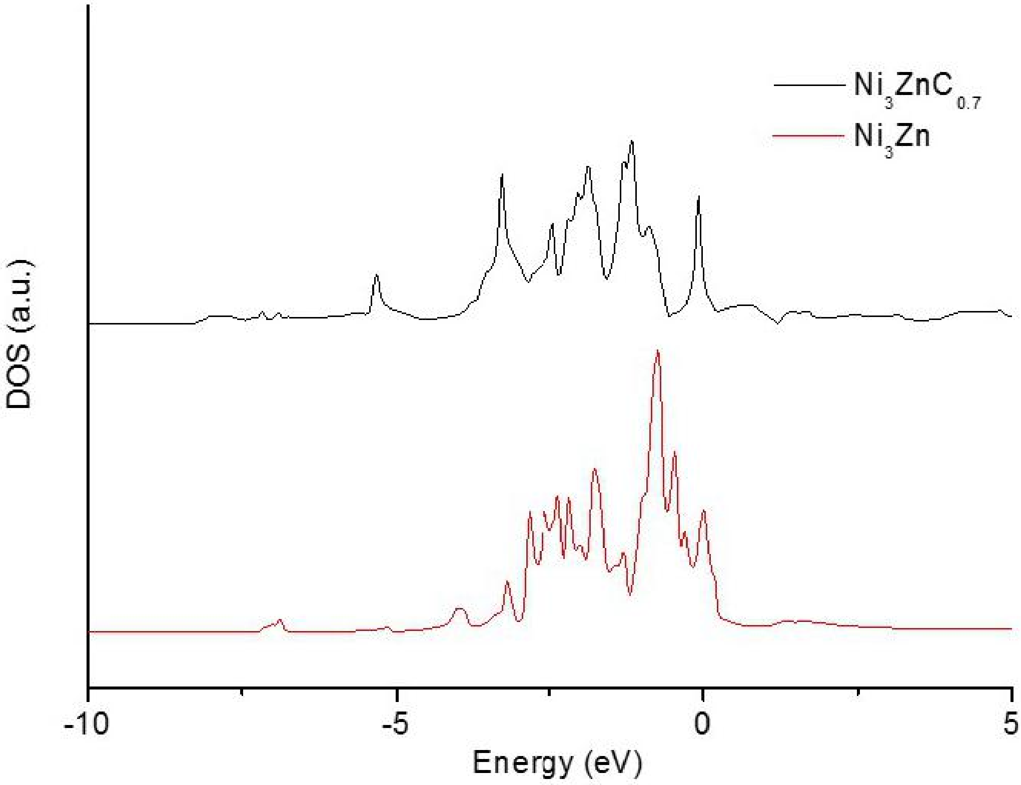

Supplementary Figure 19. The density of state of Ni_3Zn and $Ni_3ZnC_{0.7}$ structure.

Supplementary Notes

Supplementary Note 1. The desorption temperature of H_2 in Ni-based catalysts in Supplementary Fig. 10 could be divided to three parts. The low (0-200 °C) and high (above 350 °C) temperature regions, which are distributed in all of the catalysts, could be ascribed to the surface bonded H and the spillover H on the support according to previous studies[1,2], respectively. However, the identification of desorption peak appears at medium temperature region, which emerges at 315 °C for Ni and Ni_3Zn catalysts, is ambiguous. In addition, the desorption of subsurface interstitial H in Ni supported catalysts at elevated temperatures has been barely studied. Thus, based on previous theory calculation studies[3] and our experimental results, we reasonably conclude that the desorption peak emerges at 315 °C for Ni and Ni_3Zn catalysts could be assigned to interstitial H, which is absent in $Ni_3ZnC_{0.7}$ catalyst.

Supplementary Note 2. Selectivity in Supplementary Fig. 12 is calculated according to equations:

$$\mathrm{Sele}_{C_2H_4}=\left(\frac{C_{C_2H_4,out}}{C_{C_2H_2,in}-C_{C_2H_2,out}}\right)\times 100\% \quad (1)$$

$$\mathrm{Sele}_{C_2H_6}=\left(\frac{C_{C_2H_6,out}}{C_{C_2H_2,in}-C_{C_2H_2,out}}\right)\times 100\% \quad (2)$$

$$\mathrm{Sele}_{C_4H_x}=\left(\frac{2C_{C_4H_x,out}}{C_{C_2H_2,in}-C_{C_2H_2,out}}\right)\times 100\% \quad (3)$$

The C_4H_x represents the overall polymerization product which contained the 1-butene, isobutene, cis-2-Butene, trans-2-butene and butadiene.

Supplementary Note 3. Selectivity in Supplementary Fig. 16 is calculated according to equations:

$$\mathrm{Sele}_{C_2H_4}=\left(1-\frac{C_{C_2H_6,out}}{C_{C_2H_2,in}-C_{C_2H_2,out}}\right)\times 100\% \quad (4)$$

$$\mathrm{Sele}_{C_2H_6}=\left(\frac{C_{C_2H_6,out}}{C_{C_2H_2,in}-C_{C_2H_2,out}}\right)\times 100\% \quad (5)$$

$$\mathrm{Sele}_{C_4H_x}=\left(\frac{2C_{C_4H_x,out}}{C_{C_2H_2,in}-C_{C_2H_2,out}}\right)\times 100\% \quad (6)$$

The C_4H_x represents the overall polymerization product which contained the 1-butene, isobutene, cis-2-Butene, trans-2-butene and butadiene.

Supplementary Note 4. The cartesian coordinates of each atoms

(Ni_3Zn_1, model with a=b=c=3.58 Å, α=β=γ=90°)

Ni	1.789999962	1.789999962	0.000000000
Ni	0.000000000	1.789999962	1.789999962
Ni	1.789999962	0.000000000	1.789999962
Zn	0.000000000	0.000000000	0.000000000

($Ni_3Zn_1C_1$, model with a=b=c=3.66 Å, α=β=γ=90°)

Zn	0.000000000	0.000000000	0.000000000
Ni	0.000000000	1.830000043	1.830000043
Ni	1.830000043	0.000000000	1.830000043
Ni	1.830000043	1.830000043	0.000000000
C	1.830000043	1.830000043	1.830000043

Supplementary References

1. Liu, M. *et al.* Significant promotion of surface oxygen vacancies on bimetallic CoNi nanocatalysts for hydrodeoxygenation of biomass-derived vanillin to produce methylcyclohexanol. *ACS Sustainable Chem. Eng.* **8**, 6075-6089 (2020).
2. Yang, F. *et al.* Size dependence of vapor phase hydrodeoxygenation of m-Cresol on Ni/SiO2 catalysts. *ACS Catal.* **8**, 1672-1682 (2018).
3. Aleksandrov, H. A., Kozlov, S. M., Schauermann, S., Vayssilov, G. N. & Neyman, K. M. How absorbed hydrogen affects the catalytic activity of transition metals. *Angew. Chem. Int. Ed.* **53**, 13371-13375 (2014).
